# Whole genome sequence comparison of ten diagnostic brucellaphages propagated on two *Brucella abortus* hosts

**DOI:** 10.1186/s12985-015-0287-3

**Published:** 2015-04-22

**Authors:** Ekaterine Tevdoradze, Jason Farlow, Adam Kotorashvili, Natia Skhirtladze, Irina Antadze, Sophio Gunia, Nana Balarjishvili, Leila Kvachadze, Mzia Kutateladze

**Affiliations:** George Eliava Institute for Bacteriophages, Microbiology and Virology, Tbilisi, Georgia; Lugar Center for Public Health Research at National Center for Disease Control, Tbilisi, Georgia; Academic Engagement Program (AEP) Pennsylvania State University, University Park, State College, USA; Farlow Scientific Consulting Company, LLC, Lewiston, Utah USA

## Abstract

**Background:**

Recently the genome sequences of two brucellaphages, isolated in Georgia (Tb) and Mexico (Pr) were reported revealing pronounced sequence homogeneity and the presence of two major indels discriminating the two phages. Subsequent genome sequencing of six diagnostic brucellaphages: Tbilisi (Tb), Firenze (Fz), Weybridge (Wb), S708, Berkeley (Bk) and R/C phages identified three major genetic groups. However, the propensity for fine-scale genetic variability of diverse brucellaphages grown on multiple hosts within a single *Brucella* species remains unknown.

**Methods:**

We sequenced the complete genomes of ten brucellaphages following initial propagation on *B. abortus* strain 141 and after subsequent propagation on *B. abortus* strain S19. All brucellaphages were isolated and propagated at the Eliava Institute including the original Tb phage. Genomic libraries were quantified using the Qbit and sheared on the Covaris M220. QC for fragmentation was performed on the BioAnalyzer 2100. DNA libraries were prepared using an Illumina Paired-End protocol and sequenced on the Illumina MiSeq. Sequence analysis was performed using Geneious and MEGA software.

**Results:**

Comparative whole genome sequence analysis revealed genetic homogeneity consistent with previously published data as well as multiple nucleotide variations. Genomic changes as a result of passages were observed in similar genes and predominantly occurred at identical sites in separate phages. Multiple instances of within-sample genetic heterogeneity were observed often at shared genomics positions across phages. Positive selection was detected in the tail collar protein gene. We also identified a *Staphylothermus marinus* F1-like CRISPR spacer and sequences orthologous to both prophage antirepressors of *Brucella* spp. and intergenic sequences encoded by *Ochrobactrum anthropi.*

**Conclusion:**

We surveyed whole genome level diversity in phage lytic for *B. abortus* as they are propagated on alternate vaccine strains within the species. Our data extend previous results indicating select variable hotspots and broad genomic homogeneity as well as multiple common polymorphisms and within-sample variation. These data also provide additional genomes for future reference in comparative studies involving the molecular evolution and host specificity of brucellaphages.

## Introduction

Brucellosis is an important global zoonotic disease caused by members of the *Brucella* genus [1]. Members of this genus exhibit distinct primary host specificity and currently comprise ten species: *B. melitensis*, *B. suis*, *B. abortus*, *B. ovis*, *B. canis*, *B. neotomae*, *B. ceti, B. microti, B. pinnipedialis,* and *B. inopinata,* (http://www.bacterio.net/brucella.html). Typing of *Brucella* is confirmed by serum agglutination, colony morphology, CO_2_ requirement, H_2_S production, growth on thionine or basic fuchsine plates on serum dextrose agar (SDA), and diagnostic brucellaphage lysis [2,3]. Phage typing of *Brucella* is an established diagnostic and is used routinely in many diagnostic applications [4-6].

Recently, whole genome sequences of the Tbilisi phage (hereafter designated Tb_*M*_) and a brucellaphage isolated in Mexico (Pr) were reported that revealed pronounced sequence homogeneity (98.7%) and the presence of two major indels discriminating the two strains [7]. The brucellaphage genomes also encoded a prokaryotic-like DNA replication factor and a bacterial origin of replication (OriC) [7]. Many of the virion proteins show similarity to a cryptic prophage encoded by *Chelativorans* and based on amino acid similarity and gene synteny the authors suggest common ancestry between these phages that share evolutionarily related host species (*Brucella* and *Chelativorans*). Subsequent comparative whole genome analysis of six diagnostic brucellaphages including Tbilisi (Tb), Firenze (Fz), Weybridge (Wb), S708, Berkeley (Bk) and R/C phages revealed high sequence similarity and identified three major genetic groups consistent with their defined host range phenotypes [8]. Group I comprised Tb and Fz phages predominantly lytic for *B. abortus* and *B. neotomae* while Group II included Bk, R/C, and Pr phages lytic mainly for *B. abortus*, *B. melitensis* and *B. suis.* Phages Wb and S708 lytic for *B. suis, B. abortus,* and *B. neotomae* comprised Group III. The Tb phage sequenced by Flores et al was obtained from the Gamaleya Institute of Epidemiology and Microbiology, Moscow, Russia. This genome displayed comparatively greater genetic distance from all other within-group brucellaphages including an alternate Tb phage (designated Tb_*W*_) [7] obtained from the Félix d’Hérelle Reference Center in Canada. The two major indels matching those initially observed in the Tb_*M*_ and the Pr phage genomes [7] comprised the major discriminating genetic feature distinguishing major evolutionary groups of brucellaphages [8]. The distinct genetic divergence of the Tb_*M*_ genome sequence was suggested to have resulted from diversifying selection and/or genetic drift in the laboratory. The study also showed the putative phage collar protein is a hotspot for genetic variation across the major evolutionary groups and is currently undergoing positive Darwinian selection [8]. The three major evolutionary clades that comprise the evolutionary structure of brucellaphages may in-part reflect particular adaptations to differences in unique host attributes [8]. The propensity for fine-scale genetic variability of brucellaphages grown within individual *Brucella* spp. remains unknown. Such data may elucidate further the fine-scale genetic structure of this phage population and may facilitate discovery of biological determinants mediating within-species variations in host range.

In order to assess the evolutionary structure and genomic plasticity of brucellaphages following propagation on alternate host strains within the *B. abortus* species we sequenced the complete genomes of ten brucellaphages following propagation first on *B. abortus* strain 141 and after subsequent propagation on host strain S19. Phages included in this study comprised a set of ten brucellaphages lytic for *B. abortus* recently characterized at the Eliava Institute during efforts to expand the diagnostic repertoire of phages currently used in the country of Georgia. Our data revealed patterns in the genomic diversity of these phages including potential neutral and/or adaptive nucleotide polymorphisms within a set of genes that shared common sequence mutations.

## Materials and methods

### Brucellaphages and bacterial strains

#### Phage specificity and purification

All brucellaphages analyzed were originally isolated at Eliava including the original Tb phage now used worldwide (isolated from manure), 1066 (isolated from *B. canis*), 281 (*B. ovis*), 02 (*B. ovis*), 177 (*B. melitensis*), 110 (*B. melitensis*), V (*B. abortus*), 11sa (*B. suis*), 544 (*B. abortus*), 141 (*B. abortus* 141) (Table 1). Highly concentrated phages were obtained on Petri dishes (plate concentrate); purification of native virions was carried out by gradient centrifugation at 18,000G at 4°C. Briefly, Phage lysates were centrifuged at 5000 g, 4C to remove bacterial debris, then the suspension was centrifuged at 18000 g. Phage were suspended in SSC buffer (0.15 mM NaCl, 0.015 mM Na citrate, pH 7.0). Final purification of native virions was carried out by centrifugation at 18 000 g through a CsCl gradient in an SW25 rotor using a Beckman L2-65B ultracentrifuge at 4°C. Phage fractions were dialysed against SSC. Single-step growth studies were performed as described previously (Adams, 1961, p. 416). Phage were independently propagated to limit contamination on freshly prepared bacterial lawns; plaque number was determined after 24 h and 48 h of incubation at 37°C then plaque forming units (pfu) per ml were calculated. Biological properties such as adsorption, latent period, lysis time and average burst size were studied by standard methodology [9].

**Table 1:** **Reproduction parameters of phages used in this study**

**Phages**	**Isolation source**	**Time of adsorption (min)**	**% adsorption**	**Latent period (min)**	**Lysis time (min)**	**Average burst size (PFU/ml)**
Tb_141	liquid manure	190	90	200	460	40-46
Tb_19	liquid manure	180	81	210	460	50-53
110	*B. melitensis*	120	70	180-220	440	18-20
141	*B. abortus*	120	76	240	450	27-30
11sa	*B. suis*	120	53	210-240	410	28-30
1066	*B. canis*	150	98	180	425	110-120
/02	*B. ovis*	120	82	240	400	36-40
544	*B. abortus*	90-100	82	270-300	480-490	30-35
281	*B. ovis*	90-100	70	250-280	450-460	60-65
177	*B. melitensis*	180	78	240	460	40-45
V	*B. abortus*	180	37	240	480	25-30

Phage reproduction was studied on *B. abortus* 141; Tb phage alone was grown was on both, *B. abortus* S19 (indicated by Tb_19) and 141 (Tb_141) strains.

### DNA isolation, genome sequencing and assembly

#### DNA isolation and sequencing

Phage DNAs were isolated by standard procedure by phenol/chloroform extraction. Genomic libraries were quantified using the Qbit (Qbit 2.0 Fluorometer, Life technologies), sheared using the Covaris M220 focused ultrasonicator (Covaris, Inc.), and fragmented QC was performed on the BioAnalyzer 2100 (Agilent Technologies, Inc.). Libraries were subsequently modified using the Illumina Paired End Sample Prep Kit and sequenced on the Illumina MiSeq platform (Illumina, Inc.) per manufacturer’s instructions. Raw fastq files for each phage sample were exported and subjected to *de novo* assembly using CLCBio software (CLC Bio, Aarhus, Denmark) using kmer size N = 51, minimum contig size = 1000. SNP analysis, genome alignments, and Neighbor-Joining [10] dendrograms were generated using Geneious software version 6.1.5 (11). SNP loci exhibiting within-sample heterogeneity in variant calls are illustrated in Table 2 with residues comprising fractions of <50% of Illumina read populations designated in brackets. Sanger sequencing was also performed to validate SNP loci. Sequencing primers were designed from the GenBank Tb_*M*_ phage reference sequence genome for DNA sequencing on the ABI 3130×L sequencer (GenBank: NC_019446.1). Cycle Sequencing PCR was carried out using the BigDye Terminator v3.1 Cycle Sequencing Kit. For the cycle sequencing reaction, each primer was diluted to the 3.2pmol working concentration and 5-20 ng of template DNAs was used. Sequencing PCRs for each DNA sample (with single primer) was prepared in triplicate. Purification of the sequencing PCR products was performed by BigDyeX Terminator purification kit or by ETOH purification. Purified DNA products were analyzed on the ABI 3130×L sequencer (Applied Biosystems). Analysis of the sequencing data was determined using the DNASTAR- Lasergene 9.1 software (Madison, WI; http://www.dnastar.com).

### Nucleotide sequences

Whole genome sequences for all newly phage genomes in this study were deposited in GenBank: 02_19 (KJ133688), 02_141 (KJ133689), 11sa_19 (KJ133690), 11sa_141 (KJ133691), 110_19 (KJ133692), 110_141 (KJ133693), 141_19 (KJ133694), 141_141 (KJ133695), 177_19 (KJ133696), 177_141 (KJ133697), 281_19 (KJ133698), 281_141 (KJ133699), 544_19 (KJ133700), 544_141 (KJ133701), 1066_19 (KJ133702), 1066_141 (KJ133703), Tb_19 (KJ133704), Tb_141 (KJ133705), V_19 (KJ133706), V_141 (KJ133707).

In addition, arbitrary designations were used to distinguish the multiple Tb brucellaphage genomes sequenced by other institutes in addition to the Tb phage sequenced here. These include TbE, TbW, and TbM for the Tb phages grown on the S19 host strain here (KJ133704) and that by Farlow et al [8] (KC556897) and Flores et al. [7] (JN939331), respectively.

### Comparative genomics, phylogenetic structure, and selection

Whole genome alignment data using Geneious software v5.0 [11]. Unrooted Neighbor-Joining phylogenies were performed based on Jukes-Cantor model following best model fit analysis (JC) determined from Bayesian Information Criterion (BIC) in MEGA 5 [12] with boot-strapping performed using 1000 replicates. No outgroup was included as no such significant representative is currently available for the brucellaphage group. Branch lengths were measured in number of substitutions per site with bootstrap performed using 1000 replicates. The significance of testing for the presence of positive (or adaptive) selection was determined by the two tailed Z-test (P < 0.05) using the Nei and Gojobori method [13] implemented in MEGA5. The probability of rejecting the null hypothesis indicates strict-neutrality (dN = dS) in favor of the alternative hypothesis (dN > dS). Values of P less than 0.05 are considered significant at the 5% level and are highlighted. The test statistic (dN - dS) (in the Prob column) is shown in the Stat column. dS and dN are the numbers of synonymous and nonsynonymous substitutions per site, respectively. The variance of the difference was computed using the bootstrap method (1000 replicates). The analysis involved 20 nucleotide sequences. All ambiguous positions were removed for each sequence pair. There were a total of 284 positions in the final dataset.

## Results and discussion

### Phage lytic activity and host specificity

The Tb_*E*_ phage was initially cultivated on the *B. abortus* 141 (serotype I) strain originally isolated in Russia in 1950, then subsequently grown on *B. abortus* vaccine strain S19. The phenotypic characteristics of all phages are listed in Table 1. Tb phage propagated on host strain 141 phage displayed a typical slow growth cycle (190 minutes), on both host bacterial cells however adsorption time was marginally faster on vaccine strain *B. abortus* S19 (180 minutes), average burst size increased on this strain as well (50-53 PFU/cell). Other phages propagated only on the *B. abortus* 141 host strain also differed in adsorption times (Table 1). Some phages (02, 544) adsorbed on the host within two hours, while other phages displayed longer adsorption (Table 1). Average burst size was comparatively low; the highest phage counts were observed for phage 1066 (110-120 PFU/cell). Infection parameters for other brucellaphages grown on S19 were not assessed here.

### Genomic comparison and phylogenetic structure

We performed whole genome sequencing and *de novo* assembly of ten brucellaphages before and after host propagation from host strain 141 to strain S19 following 2-3 passages. This study is the first report describing whole genome level polymorphisms following serial propagation of brucellaphages on alternate *B. abortus* hosts. Following propagation on S19 each genome sequence was compared to the pre-passaged genome derived from propagation on strain 141 as well as to the brucellaphage genomes reported previously for Tb_*M*_ and Pr (Table 2). Similar genome sizes (41,149 bp), GC content (48.2%), and gene synteny were observed across the phages in this study. The Tb_*E*_ phage genome and other brucellaphage genome sequences were collinear with the published Tb_*M*_ phage genome sequence with complete synteny across all genomes analyzed. All ten phages analyzed here showed limited genomic diversity consistent with the genetic homogeneity observed between Pr and Tb_*M*_ and common restriction profiles reported previously across other brucellaphages [7,14,15]. We found close concordance in genetic similarity between the Tb_*W*_ phage [8] and the Tb_*E*_ phage sequenced in our study from the Eliava Institute that further supports the unique genetic character of the Tb_*M*_ phage [7] which appears atypical [8]. Whole genome sequencing of additional Tb phage representatives available from the Gamaleya Institute may provide further insight as to the prevalence of unique subtypes distributed by this source. Variations reported previously [8] that are specific to Tb_*M*_ alone include: at 6353 - CAAAT to ACCCG (Tb_*M*_), 11613 - TAT to ATA (Tb_*M*_), 12384 - ATATG to TATAC (Tb_*M*_), 13145 - ATCCGCTACA to CGAATAGCAC (Tb_*M*_), 13438 - GCGCGCGTAATTT to ATATATGTAATCC (Tb_*M*_), and at 17184 an insertion of residues GTAGCG. We also noted a variation unique to the Pr strain at position 34533 that displays the sequence ACCGG compared to CAATT in all other brucellaphages. We also observed partial correspondence in the content of select genes encoding mutations when compared to previously published whole genome data [8]. Genomic changes in Tb_*E*_ across propagation from host strain 141 to S19 included SNPs predominantly in ORF 21 and the phage tail collar gene at positions 15576/15578 (ORF 21) and positions 2185/22178 (Table 2). A total of five ORFs in our study (#8, 16, 21, 23, and 27) were also genetically variable across diverse brucellaphages analyzed previously [8]. In addition, a total of five ORFs (#7, 18, 43, 51, and 58) variable in previously published data [8] showed no variation among the phages analyzed in our study while seven ORFs (#12, 17, 20, 25, 26, 39, and 42) showed variability in our data set alone, in part possibly due to unique host-factor requisites unique to *B. abortus* strain 141. Overall, genome alignment comparison revealed high pairwise percent identity across all genomes as well as complete conservation of gene number and orientation. This high synteny and complete collinearity in brucellaphages of diverse origin provides further support for potential absence of recombination in brucellaphages and suggests that they may be highly adapted to potential homogenous requisites of host infection expressed by their genetically similar hosts [7,16,17]. This is in contrast to many phage populations that appear to evolve through genetic mosaicism [18,19] due to frequent non-homologous horizontal gene transfer.

**Table 2:** **Nucleotide polymorphisms in whole genome sequences of the 22 brucellaphage genomes studied**

^**a**^ **ORF**	**7**	**8**	**12**	**12**	**13**	**17**	**20**	**21**	**21**	**21**	**21**	**21**	**21**	**21**	**21**	**23**	**23**	**23**
Locus	2868	3357	7313	7799	8715	11046	13192	15576	15578	15774	15775	15996	16003	16012	16064	16700	16952	16984
Coding Δ	C/T(D)	G(R) T(L)	T(I) G(S)	A(K) C(T)	A/G(Q)	C(L) A(I)	C(T) A(N)	T(L) G(R)	C(D) A(E)	C(P) T(S)	C(S) T(L) A(H)	G(A) A(T) C(P)	T(V) GG)	A(E) C(A)	A(K) T(N)	C/T (N)	A/G (S)	C(S) A(Y)
Codon p.	3rd	2nd	2nd	2nd	1st	1st	2nd	1st	3rd	1st	2nd	1st	2nd	2nd	3rd	2nd	2nd	1st
TbM	C	G	G	A	A	C	C	G	C	C	C	G	G	C	T	C	G	C
Pr	C	G	G	A	A	C	C	G	C	C	C	G	G	C	T	C	G	C
TbW	C	G	G	A	A	C	C	G	C	C	C	G	G	C	T	C	A	C
TbE _141	T	T	G	A	A[G]	C	C	G[T]	C[A]	T	C	A	T	A	A	C	A	C
TbE _19	T	T	G	A	A[G]	C	C	G	C	T	C	A	T	A	A	C	A	C
02_141	T	T	G[T]	A	A	A	A	G	C	T	C	A	T	A	A	T	A	A
02_19	T	T	G[T]	A	A	A	A	G	C	T	C	A	T	A	A	T	A	A
11sa_141	T	G	G	A	A	C	C	G	C	C	T	G	T	A	A	C	A	C
11sa_19	T	T	T	A	A	A	A	G	C	T	C	A	T	A	A	T	A	A
141_141	T	G[T]	G	C	A	C	C	G	C	C	C	G	T	A	A	C	A	C
141_19	T	G[T]	G	C	A	C	C	G	C	C	C	G	T	A	A	C	A	C
177_141	T	T	G[T]	A	A[G]	C[A]	C[A]	G	C	T	C	A	T	A	A	C	A	C
177_19	T	T	G[T]	A	A	C[A]	C[A]	G	C	T	C	A	T	A	A	C	A	C
281_141	T	T	G[T]	A	A[G]	C[A]	C[A]	G	C	T	C	A	T	A	A	C	A	C
281_19	T	G	G	C	A	C	C	G	C	C	C[A]	C	T	A	A	C	A	C
544_141	T	G[T]	G	A	A[G]	C	C	G	C	C[T]	C	G[A]	T	A	A	C	A	C
544_19	T	G[T]	G	A	A[G]	C	C	G	C	C[T]	C	G[A]	T	A	A	C	A	C
1066_141	T	T	G	C	A	C	C	G	C	C	C[A]	G	T	A	A	C	A	C
1066_19	T	G[T]	G[T]	A[C]	A	C[A]	C[A]	G	C	C[T]	C[T]	G[A]	T	A	A	C[T]	A	C[A]
V_141	T	G	G	A	A	C	C	G	C	T	T	A	T	A	A	C	A	C
V_19	T	T	G[T]	A	A	A	A	G	C	T	C	A	T	A	A	T	A	A
110_141	T	T	G[T]	A	A	A[C]	A	G	C	T	C	A	T	A	A	T	A	A
110_19	T	T	G[T]	A	A[G]	A[C]	C[A]	G	C	T	C	T	T	A	A	C	A	C
ORF	25	26	27	27	27	27	27	27	27	27	27	39	42	43	58	NC	NC	
Locus	19780	21544	21769	21815	22178	22364	22409	22466	22469	22470	22472	28322	29512	29915	39320	40644	41105	
Coding Δ	C(P) A(H)	G (C ) T(S)	C(A) A(E)	G(D) T(Y)	G(D) T(Y)	G(D) T(Y)	G(V) T(F)	A(S) C(A)	A(K) G(A)	A(K) C(A)	A(N) T(Y)	G(L) T(M)	T(N) C(D)	C(L) A(Y)	C(I) A(Y)	NA	NA	
Codon p.	3rd	1st	3rd	1st	1st	1st	1st	1st	1st	2nd	1st	3rd	3rd	3rd	3rd	NA	NA	
TbM	C	G	C	G	G	G	G	C	A	A	A	G	T	C	A	A	C	
Pr	C	G	C	G	G	T	G	A	A	G	A	G	T	A	C	A	T	
TbW	C	G	C	G	G	T	G	A	A	C	A	G	T	C	C	T	T	
TbE _141	C	G	C	G[T]	T	T	T	A	G	C	T	G	C	C	C	T	T	
TbE _19	C[A]	G	C	G	T[G]	T	T	A	G	C	T	G	C	C	C	T	T	
02_141	C	G	C	G	G	T	G	A	G	C	T	G[T]	C	C	C	T	T	
02_19	C	G	C	G	G	T	G	A	G	C	T	G[T]	C	C	C	T	T	
11sa_141	C	G	C	G	G	G[T]	G	A	A	A	T	G	T	C	C	T	T	
11sa_19	C	G	C	G	G	T	G	A	G	C	T	G[T]	C	C	C	T	T	
141_141	C	G	C[A]	G	G	T	G	A	A	A	T	G	C	C	C	T	T	
141_19	C	G	C[A]	G	G	T	G	A	A	A	T	G	C	C	C	T	T	
177_141	C[A]	G	C	G	T	T	T	A	G	C	T	G	C	C	C	T	T	
177_19	C[A]	G	C	G	T	T	T	A	G	C	T	G	C	C	C	T	T	
281_141	C[A]	G	C	G	T	T	T	A	G	C	T	G	C	C	C	T	T	
281_19	C	T	C[A]	G	G	G[T]	G	A	A	A	T	G	C	C	C	T	T	
544_141	C	G	C	G	G	T	T	A	A[G]	A[C]	T	G	T[C]	C	C	T	T	
544_19	C	G	C	G	G	T	T	A	A[G]	A[C]	T	G	T[C]	C	C	T	T	
1066_141	C	G	C[A]	G	G	G[T]	G	A	A	A	T	G	C	C	C	T	T	
1066_19	C[A]	G	C[A]	G	G[T]	G[T]	G[T]	A	A[G]	A[C]	T	G[T]	T[C]	C	C	T	T	
V_141	C	G	C	G	G	G[T]	T	A	A	A	T	G	T	C	C	T	T	
V_19	C	G	C	G	T	T	G	A	G	C	T	G[T]	C	C	C	T	T	
110_141	C	G	C	G	G	T	G	A	G	C	T	T	C	C	C	T	T	
110_19	A	G	C	G	T	T	T	A	G	C	T	G	C	C	C	T	T	

^*a*^designates Open Reading Frame (ORF) number per the published Tb phage genome sequenced by Flores et al 2014 (JN939331).

**Figure 1:**
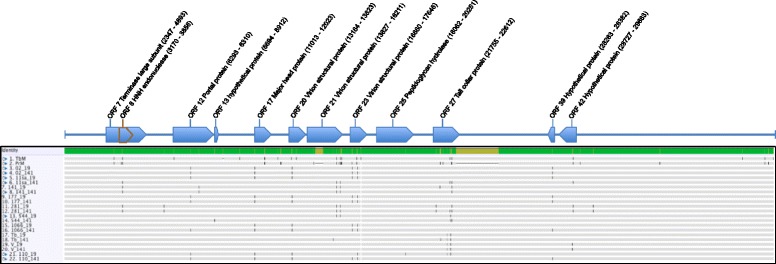
Whole genome alignments of all genomes analyzed in this study. SNPs are indicated by vertical black lines, phages are designated by phage ID number followed by underscore and propagating host strain name. Open Reading Frames number and location are illustrated above the alignment.

**Figure 2:**
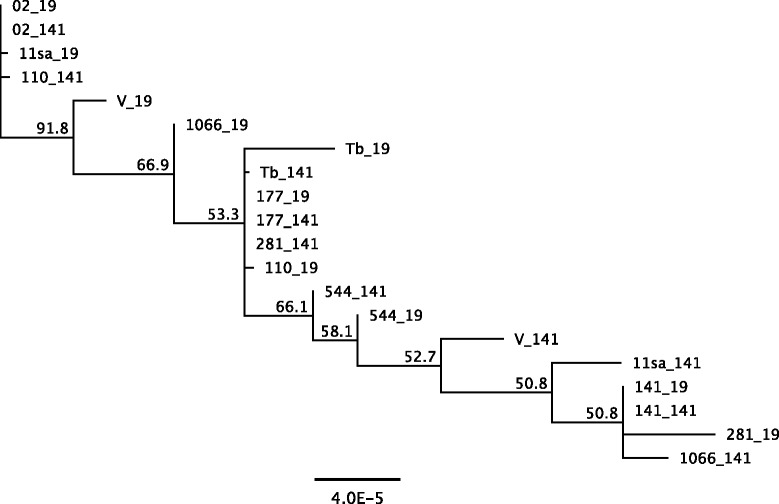
Whole genome unrooted Neighbor-Joining dendrograms for twenty brucellaphage strains based on whole genome sequences. NJ-based phylogenetic reconstructions (majority greedy clustering) comprising phage grown on *B. abortus* strain 141 and S19. Scale bars represent Nei’s genetic distance metric, values on horizontal branches represent bootstrap probabilities (1000 replicates).

The most pronounced differences previously observed between the Tb_*M*_ and Pr brucellaphages genomes include two major indel regions [7]. The first major indel region encoded by Tb extends ORF 21 that encodes a structural protein with significant orthology to hypothetical proteins in other diverse phage. The second major indel region extends the tail fiber protein (ORF 28) and encodes an additional gene (ORF29) that exhibits significant similarity to pectin-lyase-like domain containing carbohydrate binding proteins. We note the majority of variation in our study and that presented in a recent report [8] suggest this genome block encodes most of the genetic variability in brucellaphage genomes. The cellular receptor(s) used by brucellaphages have not been identified though it is possible that variations in distinct requisites of attachment across host organisms may have induced variations in tail fiber length or specificity as well as a requirement for the putative carbohydrate binding protein (ORF29). All phages in this analysis lacked the two major deletions present in the Pr phage lytic for *B. melitensis* (Figure 1). The gene variations within this region including the carbohydrate binding protein and the tail fiber protein, may reflect adaptations to host range determinants distinguishing phage lytic for *B. abortus* or *B. melitensis*, however such speculation must be experimentally confirmed. Genetic co-variations observed here shared among multiple phages lytic for *B. abortus* may represent more fine-scale adaptations occurring from propagation on a single host species, though at present this remains speculative. Phage 11sa showed the greatest variation across propagation on S19 and 141 (Figure 2). Phages 02, 141, and 177 exhibited the greatest genetic stability and showed no difference in topological placement following propagation on strain 19 while only a moderate change in topological placement was noted for phage 544 (Figure 2). While isolation of brucellaphages lytic for their host of origin is rare, resistant sub-clones have been isolated from field samples and laboratory stocks [20,21]. Previous studies on the 544 host strain reported isolation of a phage resistant descendent strain (544-FS) refractory to infection (though not adsorption) by Tb, S708 and other brucellaphages [20,21]. Resistant host strains giving rise to phage lytic for their smooth variants is a possibly explanation though this has not be reported.

### Candidate loci for host specificity

We noted a total of 24 polymorphic loci within 13 genes including two SNPs within the 3′ non-coding terminal region (Table 2, Figure 1) within the core set of Georgian brucellaphages propagated in this study. For comparison we included 11 additional loci reported previously that do not share identity with the Georgian phages analyzed here (Table 2). We noted a total of two synonomous mutations and 22 non-synonomous mutations (Table 3) within our core set of phages. Only phage 02 showed no polymorphisms on propagation from strain 141 to strain 19. Genomic changes predominantly occurred at identical sites across separate phage lineages including within-sample heterogeneity. Mixed within-sample genotypes (two-states) were observed across all loci in at least a single phage. We speculate such loci indicate emergence or maintenance of particular residues imparting a fitness cost or benefit. Though it is possible sequencing error could play some role in this variation across Illumina sequence read populations we note that in all observed sites one or both of the two alternate residues are observed in up to 100% of reads at the same individual locus in multiple alternate phages across alternate Illumina library preparations. Whether such loci indicate the potential presence of less-fit minority variants, defective virions carried over during the brucellaphage life cycle or emergence of actual quasispecies clone subsets with variable fitness must await experimental validation. In addition, several SNP loci were also observed that altered restriction site modification signatures (Table 4). Genes containing the greatest number of shared and unique polymorphisms included putative genes encoding the tail collar protein and the structural protein encoded by ORF 21 (putative neck region). Two SNP loci at positions 22364 and 22470 within the tail collar protein gene (ORF 27) in our data also displayed variability across Tb (Tb_*W*_), Firenze (Fz), Weybridge (Wb), S708, Berkeley (Bk) and R/C brucellaphages as reported previously [8]. Similar to previous data [8], the gene encoding the putative phage tail collar protein showed the most pronounced level of variation with nine coding changes comprising 27% of total SNPs among the phages analyzed. Along with the ORF 21 (structural protein), this gene is the most hyper-variable region in the genome across the brucellaphages analyzed here (Tables 2 and 3, Figure 1) and is consistent with that observed previously in other brucellaphages [8]. Three of the observed mutations in the collar protein gene resulted in stop codons and may represent premature termination of the encoded protein product. Previous speculation [8] suggested coding changes in the tail collar protein may alter the conformation/activity of the attached tail fiber protein through steric alterations or perhaps affect conformational interactions with the portal protein mediating viral DNA injection. Phage tail collar proteins assemble into ordered arrays and play a central role in binding the phage portal protein and orienting phage tail fibers [22]. Though speculative, this protein may be positioned at the nexus of both phage attachment (binding tail fiber protein) and DNA injection (phage portal protein) and could be subjected to enhanced selective forces imparted by strain-specific variations in functional requisites. For example, changes in the efficiency of genome injection (portal protein) across phages or absorption capacity (tail fibers) could select for structural modifications effecting collar organization, folding, or stability. Future experimental studies are needed to evaluate these speculations. To test whether the variability observed in the tail collar protein may be under selective pressure, we tested for the presence of positive selection acting on the collar protein gene and ORF 21 using a codon-based test of positive selection averaging over all sequence pairs. Application of the Bayesian Information Criterion (BIC) identified the Jukes-Cantor model as the best fit of the observed substitution pattern. Results were significant for positive selection acting in the tail collar gene with an overall dN-dS = 2.10 (P = 0.018). In contrast, positive selection does not appear to be acting on ORF 21 dN-dS = 1.48 (P = 0.071).

**Table 3:** **Annotated list of open reading frames showing major variations in synonomous and non-synonomous polymorphisms**

**Annotation**	**ORF#**	**Start**	**End**	**Length**	**Direction**	**Coding Δ**	**# Loci** ^**b**^	**Passage** ^**c**^
Putative HNH endonuclease	8	3,170	3,856	687	forward	S	1	N
Putative portal protein	12	6,370	8,694	2,325	forward	N	2	Y
Hypothetical protein	13	8,694	8,912	219	forward	S	1	Y
Structural protein	16	9,822	11,003	1,182	forward	N	1	N
Major head protein	17	11,013	12,023	1,011	forward	N	1	Y
Structural protein	20	13,164	13,823	660	forward	N	1	Y
Structural protein (neck motif)	21	13,827	16,211	2,385	forward	N	5	Y/N
Structural protein (peptidase_S74)^a^	23	16,660	17,646	987	forward	N	2	Y/N
Putative peptidoglycan hydrolase	25	18,062	20,251	2189	forward	N	1	Y
Structural protein	26	20,251	21,753	1,503	forward	N	1	Y
Putative tail collar protein	27	21,755	22,612	858	forward	S-N	7	Y/N
Hypothetical protein	39	28,262	28,382	121	reverse	N	1	Y
Hypothetical protein	42	28,727	29,683	957	reverse	S-N	2	Y

^a^Ortholog encoded by Brucella 83/13.

^b^Indicates number of variable locations oberved within individual ORFs.

^c^Indicates loci showing polymorphism among passaged strains (Y) versus variants present only within the TbM and Pr reference strains (N).

**Table 4:** **SNP loci resulting in restriction site modifications**

**Restriction sites**																
	**HaeII**	**HhaI**	**Cfol**	**AluI**	**HinfI**	**MseI**	**HaeIII**	**TaqI**	**SalI**	**HincII**	**AccI**	**MspI**	**HpaII**	**MboI**	**Sau3AI**	**NdeI**
3357	G	G	G	-	-	-	-	-	-	-	-	-	-	-	-	-
11046	-	-	-	a	A		-	-	-	-	-	-	-	-	-	-
13192	-	-	-	-	-	A	-	-	-	-	-	-	-	-	-	-
15576-8	-	-	-	-	-	-	-	t	t	t	t	-	-	-	-	-
15774	-	-	-	-	-	-	C	-	-	-	-	-	-	-	-	-
15775	-	-	-	-	-	T	-	-	-	-	-	-	-	-	-	-
15996	-	G	G	-	-	-	-	-	-	-	-	C	C	-	-	-
19780	-	-	-	-	-	-	a	-	-	-	-	-	-	-	-	-
22178	-	-	-	-	-	-	-	-	-	-	-	-	-	t	t	-
22470	-	-	-	-	-	-	-	-	-	-	-	-	-	-	-	a
28322	-	-	-	-	-	-	t	-	-	-	-	-	-	-	-	-

Note: SNPs in upper case letters designate generation of restriction sites, lower case letters designate abolished restriction sites.

We also observed common mutations within the gene encoding the putative brucellaphage HNH endonuclease (ORF 8, R63L) (Table 2, Figure 1). HNH endonucleases are homing endonucleases utilized in DNA binding, nicking and degradation, of viral and bacterial nucleic acids. The HNH catalytic domain of phage and bacterial homing endonucleases consist of the conserved catalytic residues His-Asn-His as well as a zinc-binding site [CxxC] and a zinc ribbon (ZR) domain with potentially multiple zinc-binding sites ([CxxxxC], [CxxxxH], [CxxxC], [HxxxH], [CxxC] or [CxxH]) present at the N-terminus or C-terminus [23]. These proteins require divalent transition metals for endonuclease activity and variations in zinc binding mediate differences in enzymatic activity. In brucellaphage, it is possible this protein functions as a replication initiation protein nicking phage dsDNA during genome replication. In our analysis, phages Tb_*M*_, Pr, 11sa-141, 141-141, 141-19, and 281-19 possess an arginine at coding position 63 while all other phage including Tb encode a leucine at this position. The location of the active site within the brucellaphage HNH endonuclease protein lies between residues 160 to 184 within the ZR domain and is flanked by two potential zinc-binding sites (data not shown). The variable site R63L is located within this domain flanked by multiple potential zinc binding sites within the ZR domain. Furthermore, in contrast to the flanking position of genes encoding terminase subunits and HNH endonucleases (within 5 ORFs) previously observed in tailed Kala et al [24], we note that the non-tailed brucellaphages, encode the HNH endonuclease gene overlapping the mid-portion of the large subunit terminase gene. This finding further supports a potential functional association between these proteins as well as both the conservation of terminase associated HNH proteins and existence of a potentially distinct functional subfamily of the HNH endonucleases noted previously by Kala et al [25]. The observed SNP at position 3357 that results in a putative R63L mutation in the HNH endonuclease is silent in the putative large terminase subunit protein. The putative intein-encoded HNH endonuclease gene located out of frame within the large terminase subunit is present in all annotated brucellaphage genomes published to date (7, 8). Inteins are parasitic genetic elements that often encode HNH endonucleases to confer mobility. Sequence variation within these genes across brucellaphage host range variants have been previously noted (7,8). In our analysis, sequence similarity searches did not reveal signatures of other parasitic genetic elements in this region, such as group I and Group II introns. We speculate that the sequence variation in the intein-encoded HNH endonuclease gene and the terminase may represent stochastic variation, or, result from fine-scale differences in the biological properties of these proteins during phage replication within the bacterial host milieu. Such factors may include parameters effecting the evolutionary maintenance of the intein-HNH parasitic element such as intein homing efficiency and target nucleotide binding, DNA cleavage and splicing, or co-factor binding specificity. In any case, future experimental investigation may identify the role, if any, of the observed sequence variation.

Comparative sequence analysis also revealed a sequence comprising 299 bp residues (26701-27600; Tb_*M*_) spanning two hypothetical proteins that showed significant similarity to a region directly upstream of a phage integrase motif in the genome of *Ochrobactrum anthropi.* This orthologous sequence shared between brucellaphage and *O. anthropi*, is interesting as *O. anthropi* is the closest known relative to the Brucellaceae [25,26]. It is postulated that major clades in the Brucellaceae family that now maintain an intracellular lifestyle evolved from *Ochrobactrum*-like relatives free living in soil and plant-associated rhizosphera. The phage-related *Ochrobactrum*-like motif observed in the genome of brucellaphages may reflect common ancestry between phages infecting these distantly related bacterial hosts though with low evolutionary signal due to the likely divergent selective pressures separating their current niches. In addition, a 36 bp residue sequence at the 3′ end of this region (27218-27253) displayed significant similarity to a prophage antirepressor encoded by *B. canis* strain HSK A52141. This sequence is also shared broadly across other *Brucella* host species. Further BLAST analysis also revealed a motif comprising 27 nucleotide residues at the 3′ terminus of all phages showed significant similarity to a single CRISPR spacer sequence in the CRISPR database (http://crispr.u-psud.fr/crispr/) encoded by *Staphylothermus marinus* F1. This orthologous sequence shared between brucellaphage genomes and the *S. marinus* CRISPR spacer showed no significant similarity to other available sequences in GenBank. Interestingly, phage resistance systems such as CRISPRs are notably absent in *Brucella* genomes with the exception of questionable CRISPR-like genetic structures in select *B. melitensis* and *B. pinnipedialis* strains (http://crispr.u-psud.fr/crispr/).

## Conclusions

We surveyed whole genome level diversity in phage lytic for *B. abortus* and revealed fine-scale patterns in the genetic structure of these phages as they are propagated on vaccine strains within this species. Previous studies showed the relatively homogenous genomes of brucellaphages derived from diverse *Brucella* species express frequent polymorphisms in the phage collar protein that may reflect host selection. Our data extend these results confirming such diversity on a finer scale during propagation within the *B. abortus* species and illustrate multiple common sequence variations across similar genes that frequently display within-sample genetic heterogeneity. These data also provide a comparison of candidate loci for potential genotype-phenotype associations that can be explored in the Tb phage model while also yielding additional genomes for future reference in comparative studies involving the molecular evolution of brucellaphages.
